# A systematic review of anti-obesity medicinal plants - an update

**DOI:** 10.1186/2251-6581-12-28

**Published:** 2013-06-19

**Authors:** Shirin Hasani-Ranjbar, Zahra Jouyandeh, Mohammad Abdollahi

**Affiliations:** 1 Obesity & Eating Habits Research Center, Endocrinology & Metabolism Research Institute, Shariati Hospital, Tehran University of Medical Sciences, Tehran, Iran; 2 Endocrinology & Metabolism Research Center, Endocrinology & Metabolism Research Institute, Tehran University of Medical Sciences, Tehran, Iran; 3 Faculty of Pharmacy, and Pharmaceutical Sciences Research Center, Tehran University of Medical Sciences, Tehran, Iran

**Keywords:** Herbal medicine, Obesity, Systematic review

## Abstract

Obesity is the most prevalent health problem affecting all age groups, and leads to many complications in the form of chronic heart disease, diabetes mellitus Type 2 and stroke. A systematic review about safety and efficacy of herbal medicines in the management of obesity in human was carried out by searching bibliographic data bases such as, PubMed, Scopus, Google Scholar, Web of Science, and IranMedex, for studies reported between 30th December 2008 to 23rd April 2012 on human or animals, investigating the beneficial and harmful effects of herbal medicine to treat obesity. Actually we limited our search to such a narrow window of time in order to update our article published before December of 2008. In this update, the search terms were “obesity” and (“herbal medicine” or “plant”, “plant medicinal” or “medicine traditional”) without narrowing or limiting search items. Publications with available abstracts were reviewed only. Total publications found in the initial search were 651. Total number of publications for review study was 33 by excluding publications related to animals study.

Studies with Nigella Sativa, Camellia Sinensis, Crocus Sativus L, Seaweed laminaria Digitata, Xantigen, virgin olive oil, Catechin enriched green tea, Monoselect Camellia, Oolong tea, Yacon syrup, Irvingia Gabonensi, Weighlevel, RCM-104 compound of Camellia Sinensis, Pistachio, Psyllium fibre, black Chinese tea, sea buckthorn and bilberries show significant decreases in body weight. Only, alginate-based brown seaweed and Laminaria Digitata caused an abdominal bloating and upper respiratory tract infection as the side effect in the trial group. No other significant adverse effects were reported in all 33 trials included in this article.

In conclusion, Nigella Sativa, Camellia Synensis, Green Tea, and Black Chinese Tea seem to have satisfactory anti-obesity effects. The effect size of these medicinal plants is a critical point that should be considered for interpretation. Although there was no report for side effect in these trials, we believe that safety of these plants still remains to be elucidated by further long-term studies.

## Introduction

Obesity is becoming one of the most prevalent health concerns among all populations and age groups worldwide, resulting into a significant increase in mortality and morbidity related to coronary heart diseases, diabetes type 2, metabolic syndrome, stroke and cancers [1–3]. Prevention and treatment of this problem are an important deal for health systems, whose aim is to reduce the obesity and overweight prevalence, and related complications over the world [4]. Both lifestyle and pharmacotherapy interventions have been considered by physicians and other health care professionals as obesity treatment modalities. Studies show that only 5-10% subjects can maintain their weight loss over the years [5]. The complex pathogenesis of obesity indicates the need of different intervention strategies to confront this problem with a simple drug therapy which is more acceptable to patients [4]. Disappointing results, after cessation the lifestyle modification or pharmacotherapy indicated the need of other treatment modalities to produce better and long-lasting results, in terms of weight loss [6]. Herbal supplements and diet-based therapies for weight loss are among the most common n complementary and alternative medicine [CAM] modalities [7]. A vast range of these natural products and medicinal plants, including crude extracts and isolated compounds from plants can be used to induce weight loss and prevent diet-induced obesity. In the recent decades, these have been vastly used in management of obesity [4, 8] due to containing a large variety of several components with different anti-obesity and anti-oxidant effects on body metabolism and fat oxidation. Medicinal plants have been investigated and reported to be useful in treatment of obesity, diabetes and other chronic diseases [9, 10].

To date, some reviews on anti-obesity agents have been accomplished including, our systematic review on efficacy and safety of herbal plants in the treatment of obesity that published 4 years ago [11]. Because of the increasing number of randomized clinical trials conducted in the recent years, we felt the need for a new systematic review on this topic with a special focus on clinical trials. Therefore, the aim of the present review was to update data on potential anti-obesity herbal plants, and review the scientific data, including experimental methodologies, active components, and mechanisms of action against obesity in human.

## Methods

PubMed, Scopus, Google Scholar, Web of Science, and IranMedex databases were searched for studies reported between 30th December 2008 to 23rd April 2012 on human or animals investigating the benefits and harms of herbal medicines to treat obesity. The search terms were “obesity” and (“herbal medicine” or “plant”, “plant medicinal” or “medicine traditional”) without narrowing or limiting search items. Publications with available abstracts were reviewed. The main outcome measures were defined as body weight, body fat, including fat mass/fat weight or fat percentage/visceral adipose tissue weight, waist or hip circumference, triceps thickness and appetite, and the amount of food/energy intake.

Abstracts of publications on human studies with the main outcome as mentioned above were included. In vitro studies, review articles and letters to the editor were excluded. The articles were reviewed for abstracts and title by two reviewers. Due to our inclusion and exclusion criteria, the duplicate articles were eliminated.

## Results

### Body weight

Significant decrease in body weight was seen by Nigella Sativa, Camellia sinensis, Crocus sativus L, seaweed laminaria digitata, Xantigen, virgin olive oil, Catechin enriched green tea, Monoselect Camellia, Oolong tea, Yacon syrup, Irvingia Gabonensi, Weighlevel, RCM-104 compound of Camellia, Sinensis, Pistachio, Psyllium fibre, black Chinese tea, sea buckthorn and bilberries.

### Body fat

Significant decrease in body fat was seen by Xantigen [16], Catechin-enriched green tea [18], Irvingia gabonensis a West African plant [22], RCM-104 a compound of Camellia Sinensis, Semen Cassiae and Flos Sophorae [23], Psyllium Fibre [25], and black Chinese tea [Pu-Erh tea] [26]. Oolong tea showed a decrease in subcutaneous fat content not total body fat [20]. Debese showed a reduction in triceps skin folds in a trial [28].

### Waist and hip circumference

There was a significant decrease in waist and hip circumferences with Nigella Sativa [12], Xantigen [16], Catechin enriched green tea [18], Yacon Syrup [21], Irvingia gabonensis [22], Debese [28], Whole grain [29], Lycium barbarum [30], black Chinese Tea [26], Sea buckthorn, and bilberries [27]. Monoselect Camellia from green tea extract reduced the waistline only in men [19]. Pu’er tea [black Chinese tea] decreased the waist-hip ratio significantly [31].

### Food intake

A significant decrease in appetite was shown in trials by Trigonella Foenum-graecum L. [32], Fungreek fiber [33]. An extract of Blueberry Bioactives [34], Epigallocatechin of green tea [35], Northern Berries [36], alginate-based brown seaweed Laminaria Difitata [15], and RCM-104 compound of Camellia Sinensis [23] did not show any relevant decrease in appetite.

### Other effects

Anti-hyperglycemic, anti-hyperlipidemic, and anti-oxidant effects were detected in these trials [see Table 1].

**Table 1:** **Human studies considering herbal medicines for treatment of obesity**

Authors	Target	Herbs [scientific name]	Study	Dose/Duration	Groups	Main outcome	Other relevant effects & complications	Weight Before/Placebo	Weight After/Treatment	P-value
Datau et al 2010 [12]	Obese male [n = 50]	*Nigella sativa*	RCT [double blind]	Two Cap of 750 mg *NS* twice daily/3 mo	I: extract C: flour	Very Sig. reduction of BW, WC, and SBP	Non-Sig. reduction in serum free testosterone, DBP, FBS, TG and HDL-chol, uric acid, hs-CRP, and non-Sig. increase of adiponectin	77.11 ± 4.86	72.60 ± 5.41	0.000
Stull et al 2010 [34]	Obese, non-diabetic & insulin resistant [n = 32]	Blueberry composed of [*Tifblue Vaccinium ashei* and *Rubel Vaccinium corymbosum*].	RCT [double blind]	22.5 g BB twice daily/6 wk	I: extract C: placebo	Sig. improvement in insulin sensitivity, No Sig. changes in adiposity, energy intake, and inflammatory biomarkers	-	98.7 ± 3.1	99.1 ± 3.1	NS
Godard et al 2010 [49]	Obese, pre-diabetic [n = 29]	*Opuntia ficus*-*indica*	RCT [double blind]	200 mg/16 wk	I: extract C: placebo	Statistically Sig. decrease in blood glucose concentration, no Sig. changes in body composition variables	Non-Sig. changes in blood chemistry parameters [insulin, proinsulin, hs-CRP, adiponectin, HbA1C]	107.13 ± 22.24	108.39 ± 24.90	NS
Basu et al 2011 [13]	Obese with Metsyn [n = 35]	*Camellia Sinensis*	RCT	4 cups/d green tea or 2cap and 4 cups water/d/8 wk	I: green tea or extract C: water	Sig. decrease in BW and BMI	A decreasing trend In LDL-chol and LDL/HDL ratio	96.4 ± 4.7	94.5 ± 4.5	0.28
Gout et al 2010 [14]	Mildly over wt. women [n = 60]	Satiereal, [*Crocus sativus L* extract]	RCT [double blind]	176.5 mg/d/8 wk	I: extract C: placebo	A Sig. BW reduction	Decrease in mean snacking frequency	Placebo 73.9 ± 1.7	Treatment 73.2 ± 1.1	0.72
Thielecke et al 2010 [35]	Obese male [n = 10]	Epigallocatechin-3-gallate [EGCG] of green tea	RCT [double blind]	Low EGCG 300mg, high EGCG 600mg/3 d	I: EGCG [low/ high + caffeine], caffeine C: placebo	Increase in fat oxidation	-	-	-	-
Mirmiran et al 2010 [50]	Hyperlipidemic [n = 51]	Pomegranate seed oil [PSO]	RCT [double blind]	400 mg PSO twice daily/4 wk	I: extract C: placebo	Unchanged body composition variables, decreased TG and the TG: HDL-chol ratio	Unchanged Serum TC, LDL-chol and glucose concentrations	(No significant change in BMI seen)	-	-
Lehtonen et al 2010 [36]	Healthy women volunteers [n = 61]	Northern berries	RCT	163 g/20 wk	I: extract C: control	Non-Sig. change in calorie intake, WC, increase in plasma adiponectin level	High decrease in the ALAT value, no change in HOMA-IR, Fasting plasma insulin, fasting plasma TC, TG, hs-CRP, TNF-α or ORAC, small increase in fasting plasma HDL	81.7	81.9	NS
Egert et al 2010 [51]	Obese [n = 93]	Quercetin	RCT [double blind]	150 mg/d/6 wk	I: extract C: control	No change in nutritional status [BW, WC, fat mass, fat-free mass]	Decreased serum HDL- chol, apoA1, increased the LDL/HDL ratio	-	-	-
Chevassus et al 2010 [32]	Healthy over wt. male volunteers [n = 39]	*Trigonella foenum*-*graecum L*.	RCT [double blind]	1176 mg [approximately 14 mg.kg-1]/d /6 wk	I: extract C: control	Decrease daily fat consumption, Non-Sig. effect on wt., Appetite/satiety scores or oxidative parameters	Decrease in the insulin/glucose ratio	-	-	-
Gurrola-Díaz et al 2010 [52]	Human	*Hibiscus sabdariffa*	RCT	100 mg/d [1.4 mg/kg]/1 mo	I: HSEP, HSEP + preventive diet [±metsyn] C: Preventive diet	Sig. reduced glucose and TC levels, increased HDL-chol levels	-	-	-	-
Odunsi et al 2009 [15]	Obese [n = 48]	Alginate based on brown seaweed *Laminaria digitata*	RCT	6 Cap per d /10 d	I: compound C: placebo	No effect on gastric motor functions, satiation, appetite, or gut hormones	Abdominal bloating, upper respiratory tract infections	-	-	-
Abidov et al 2010 [16]	Obese, premenopausal women [n = 151]	*Xanthigen* [brown marine algae fucoxanthin + pomegranate seed oil [PSO]]	RCT [double blind]	600/2.4 mg, 400/1.6 mg /16 wk	I: Extract C: control	Sig. reduction of BW, WC, body and liver fat content	Reduction in liver enzymes, serum TG and CRP, increase REE in NAFLD	92.5 ± 1.5	88.2 ± 1.9	<0.05
Razquin et al 2009 [17]	Human [n = 187]	Virgin olive oil, nuts	RCT [Randomized dietary trial : PREDIMED trial]	3 yr	I: Mediterranean diet C: control	Reduction in BW	Higher levels of plasma total antioxidant capacity	71.98 ± 11.59	78.46 ± 12.11	0.015
Wang et al 2009 [18]	Moderately over wt. [n = 182]	Catechin enriched green tea	RCT	458 mg, 468 mg, 886 mg /90 d	I: extract C: Placebo	Decrease in estimated intra-abdominal fat [IAF] area, in WC, BW, reduction in total body fat		69.8 ± 9.1	69.9 ± 12.1	<0.05
Di Pierro et al 2009 [19]	Obese [n = 100]	*Monoselect Camellia* [containing green tea extract: GreenSelect Phytosome]	RCT	150 mg/90 d	I: hypocaleric diet + extract C: hypocaleric diet	Sig. wt. loss and decreased BMI	Reduce leptin, reduce waistline only in men, decrease TC & TG levels	96.142 ± 18.012	82.298 ± 15.326	<0.001
Qidwai et al 2009 [53]	Human [n = 123]	*Nigella Sativa*	RCT [double blind]	-	I: extract C: placebo	Non-Sig. change in BW	Non-Sig. change in serum lipid levels, BS, BP	-	-	-
Mathern et al 2009 [33]	Healthy obese [n = 18]	Fenugreek fiber	RCT [Single blind]	4 or 8 gr/3.5 hr	I: extract C: control	Increased mean ratings of satiety and fullness, reduced ratings of hunger and prospective food consumption, reduce energy intake	No difference for AUC blood glucose, increase in AUC for insulin levels	-	-	-
He et al 2009 [20]	Diet induced obese or over wt. [n = 102]	Oolong tea	RCT	8 g/6 wk	I: extract C: control	Sig. decrease in BW, decrease in subcutaneous fat content	Decrease TC & TG plasma levels	74.1 ± 8.2	71.2 ± 8.1	<0.05
Genta et al 2009 [21]	Obese & slightly dyslipidemic pre-menopausal women	Yacon syrup	RCT [double blind]	0.29 g and 0.14 g Fructo-oligosaccharides/kg/d/120 d	I: extract C: control	Sig. decrease in BW, WC, BMI	Decrease in fasting serum insulin and HOMA-IR, increased defecation frequency and satiety sensation	91.2 ± 8.4	76.2 ± 6.1a	<0.05
Ngondi et al 2009 [22]	Over wt. &/or obese [n = 102]	West African Plant [*Irvingia gabonensis*]	RCT	150 mg /10 wk	I: extract C: placebo	Sig. improvements in BW, body fat, and WC	Sig. improvements in plasma TC, LDL-chol, BS, CRP, adiponectin and leptin levels	97.9 ± 9.1	85.1 ± 3.1	<0.01
Snitker et al 2009 [41]	Healthy [n = 80]	Capsinoids	RCT [double blind]	6 mg/d/12 wk	I: powder C: placebo	Decrease in abdominal adiposity, non-Sig. change in REE, higher fat oxidation, No difference in overall percentage body fat	-	(Weight change) –0.49 6 2.37	–0.92 6 3.12	0.86
Rehman Riaz et al 2011 [28]	Obese [ n = 100]	Debese	RCT	2 yr	I: Debese C: Sibutramine	Decrease in BMI, WC	Reduction of triceps skin fold	-	-	-
Omar Said et al 2011 [54]	Healthy [n = 66]	Weighlevel [The leaves of lady’s mantle, olive and wild mint, the seeds of cumin]	RCT	310 mg tablet^−1^ [containing: 60 mg A. Vulgaris L., 50 mg O. europaea L., 20 mg Mentha longiforiaL., 25 mg C. cyminumL., 7 mg vitamin C and 148 mg tricalciumphosphate]/before each meal/3 mo	I: Tablets [before each meal] C: Tablets [before just 3 main meals]	Reduced BMI, Sig. and progressive wt. reduction	No Minor or major adverse effect	90.5 ± 1.2	78.5 ± 1.4	<0.0005
Lenon et al 2012 [23]	Obese [n = 117]	RCM-104: Compound of *Camellia Sinensis* [*Lu Cha Ye*—Green tea], *Semen Cassiae* [Jue Ming Zi], and Flos Sophorae [Huai Hua].	RCT [double blind]	500 mg granule extract 4 Cap per time, 3 times per d /12 wk	I: extract C: placebo	Reduced wt., BMI and body fat, non-Sig. changes in food intake	Sig. improvements in quality of life of participants	99.5 ± 15.1	98.0 ± 15.4	0.002
Venn et al 2010 [29]	Healthy volunteers [n = 113]	Whole grain	RCT	2 serves of pulses and 4 serves of wholegrain foods per d/18 mo	I: wholegrain C: control	No Sig. wt. loss	Decreased WC	100 ±20.7	94 ±22.8	NS
Direling et al 2010 [55]	Over wt. & obese [n = 130]	Pine bark	RCT [double blind]	200mg/d/12 wk	I: extract tablets C: placebo	No Sig. change in BMI	Non-Sig. change in levels of insulin, lipid profile, FBS and lipoprotein chol particle size, liver transaminase test results, high-sensitivity CRP and BP	-	-	-
Li et al 2010 [24]	Human [n = 59]	Pistachio	RCT	53g/12 wk	I: pistachio C: pretzels	Wt. loss, reduced BMI	Lower TG levels	86.0 6 1.4	82.3 6 1.6	<0.01
Pal et al 2011 [25]	Over wt. & Obese	Psyllium Fibre	RCT	12 wk	I: healthy diet + fibre, fibre C: placebo, healthy diet + placebo	Significant decrease in wt., BMI & %total body fat	Reduction in TG, insulin,TC & LDL-chol	-	-	<0 · 001
Amagase et al 2011 [30]	Healthy over wt.	*Lycium barbarum*	RCT	30, 60, and 120 ml/14 d	I: extract C: control	Increase of postprandial energy expenditure, Sig. decrease in WC	Increase in metabolic rate	-	-	-
Kubota et al 2011 [26]	Pre-obese male [n = 36]	Black Chinese [Pu-Erh] tea [BTE]	RCT [double blind]	333 mg before each of 3 daily meal/12 wk	I: extract C: control	Decrease in BW and BMI, Sig. effects in reducing the mean WC and visceral fat values	No adverse effects	-	-	<0.05
Chu et al 2011 [31]	Human with Metsyn [n = 90]	Pu'er tea	RCT [double blind]	4 Cap each time, twice per d/3 mo	I: extract C: placebo	Decrease in BMI, waist-hip ratio	Decreased fasting and 2 h postprandial blood glucose, serum TC, TG, LDL-chol and apolipoprotein B-100	-	-	-
Lehtonen et al 2011 [27]	Over wt. & obese women [n = 80]	Sea buckthorn [SB], and bilberries [BBs]	Comparative study	followed four different berry diets with wash out periods: [BB, SB, SB phenolic extract [SBe] and SB oil [SBo]/33-35 d	-	Sig decrease in WC after BB and SB periods and also a small decrease in BW after BB diet	Vascular cell adhesion molecule decreased significantly after BB and SBo periods, and in intercellular adhesion molecule [ICAM] after SBe diet	-	-	-

Abbreviations: *NS* Nigella Sativa, *ALAT* Alanine aminotransferase, *HSEP* Hibiscus sabdariffa extract powder, *TC* Total Cholestrol, *TG* Trigelycerides, *BMI* Body Mass Index, *RCT* Randomized Clinical Trials, *Sig* Significant, *WC* Waist Circumference, *BW* Body weight, *BP* Blood Pressure, *SBP* Systolic Blood Pressure, *DBP* Diastolic Blood Pressure, *BS* Blood Sugar, *FBS* Fasting Blood Sugar, *Chol* Cholestrol, *Cap* Capsule, *Metsyn* Metabolic syndrome, *ORAC* oxygen radical absorbance capacity, *REE* Resting Energy Expenditure, *NAFLD* non-alcoholic fatty liver disease, *AUC* Area Under the Curve, *HDL-chol* High density lipoprotein cholesterol, *LDL-chol* Low density lipoprotein cholesterol, *HOMA-IR* homeostasis model assessment-estimated insulin resistance index, *ICAM* intercellular adhesion molecule, *d* day, *mo* month, *wk* week, *yr* year, *hr* hour.

### Adverse effects

Only alginate-based brown seaweed Laminaria digitata caused an abdominal bloating and upper respiratory tract infection as a side effect in the trial group [15]. There were no other significant adverse effects reported in all 33 trials included in this article.

## Discussion

Many studies reported the anti-obesity effects of different herbal plants containing minerals or chemical extracts of plants. All herbal plants with anti-obesity effects are summarized in Table 1 with information of their active components and effects on the body. Anti-obesity effects such as decreasing body weight, body mass index or waist circumference in humans was seen in most of these studies. Some of them showed an anti-obesity effect by decreasing total body fat [16, 18, 20, 22, 23, 25, 26, 28].

A study showed a significant decrease in body weight by *Cissus Quadrangularis* (CQ), *Sambucus Nigra*, *Asparagus Officinalis*, *Garcinia Atroviridis*, Ephedra and Caffeine, Slimax (extract of several plants, including *Zingiber officinale* and Bofutsushosan) [11]. In this study, the effect of Epigallocatechin-3-gallate in combination with caffeine was evaluated, with no important changes in body weight or energy expenditure. However, anti-obesity effects of green tea components were reported in many trials.

Anti-obesity mechanisms for herbal plants included reduction in lipid absorption, reduced energy intake, increased energy expenditure, decreased pre-adipocyte differentiation and proliferation, or decreased lipogenesis and increased lipolysis [37]. Decreased energy intake from the gastrointestinal tract is caused by distinct types of tea [e.g. green, oolong, and black tea] acting on pancreatic lipase. In this review, weight loss by different tea components containing catechin and epigallocatechin-3-gallate polyphenols isolated from unlike kinds of teas was observed [18, 26, 35]. Polyphenols of different types obtained from tea extracts (e.g. L-epicatechin, epicatechin-3-gallate, epigallocatechin, epigallocatechin-3-gallate), showed strong inhibitory activity against pancreatic lipase, which led to weight loss [38, 39].

Nigella Sativa showed a significant weight loss and reduced waist circumference with a mild reduction in fasting blood sugar, triglycerides and low-density lipoprotein levels [12]. Pistachio [24], Psyllium Fibre [25], black Chinese Tea [26], Camellia Sinensis [23], Yacon Syrup [21], Oolong Tea [20], Xantigen [16] and olive oil [17] showed the same effects on the body. A systematic review on medicinal plants useful in diabetes mellitus showed that some herbal plants possess anti-hyperlipidemic effects, and this property is statistically significant in the treatment of obesity [40].

Some components affect body weight by changes in body-fat metabolism and oxidation or increasing metabolic rate, which was shown in trials by Epigallocatechin-3-gallate of green tea [35], virgin olive oil [17], Capsinoids [41] and Lycium Brbarum [30] causing a higher fat oxidation in human. These compounds act by activating lipid metabolism, acceleration of oxidation, suppression of fatty acid synthesis and PPARc agonistic activity [37].

A systematic review done on potential herbal sources effective in oxidant-related diseases showed some potential of some plants like Nigella sativa and green tea to decrease lipid peroxidation in plasma or liver, which seem a mechanism of anti-obesity effect. Higher anti-oxidant and anti-obesity activity was shown by green tea due to its high concentration of catechins, including epicatechin (EC), epicatechin-3-gallate (ECG) and epigallocatechin-3-gallate (EGCG) [13, 18, 23]. The anti-oxidative role of herbal plants in different kinds of human diseases, such as diabetes mellitus, obesity and hyperlipidemia has been already reported in literature [40, 42–44]. Those articles focused on herbal plants effective on obesity while lifestyle changes or dietary regimens were not included. However whole grain, pistachio, virgin olive oil and nuts were investigated solely and found efficient in reduction of obesity [17, 24, 29].

The alginate-based brown seaweed Laminaria Digitata [15] caused abdominal bloating and upper respiratory tract infections as a side effect but no other studies reported the same adverse effect.

In the included studies, only few has reported adverse effects, but it is notable that some kind of adverse effects may only happen when drugs used in higher sample size or when approved for marketing widely. Therefore, we cannot conclude that use of these herbals is without adverse effects. We believe that safety of these plants remains to be elucidated by further long-term studies.

## Conclusion

Different methods have been used to reduce body weight and its complications for many years. Disappointing results after cessation the lifestyle modification or pharmacotherapy compelled the researchers and physicians to rethink to find a new, safe, and striking therapeutic alternative for this global health concern. Herbal medicines have been in attention as an effective option to reduce body weight and body fat. Taking all results collectively, Nigella sativa, Camellia synensis, green tea, and black Chinese tea were found to have acceptable anti-obesity effects. Furthermore, there have been some reports on anti-oxidative stress effects of some of these plants which may be important in the management of other diseases accompanying with obesity like cardiovascular diseases and diabetes [9, 45]. By now, only one anti-obesity drug called orlistat have been approved by the US food and drug administration for long-term treatment in obese patients. Recent researches show different medications having anti-obesity effects by several mechanisms, including exenatide a glucagon-like peptide [GLP] acting as an incretin hormone [46], Lorcaserin a novel selective serotonin 2C (5-HT2C) receptor agonist that modulates food intake in hypothalamus [47] and PYY 3–36 and oxyntomodulin, a glucagon-like peptide 1(GLP-1) receptor agonist that regulate food intake [48]. The need to discover anti-obesity drugs having better efficacy and lower adverse effect is still felt. The results of this kind of studies can be helpful for pharmaceutical industries to study on the components of these herbs and investigate further to find a mixture of those components with higher efficacy. Furthermore, further well-designed clinical trials are still needed to focus on both safety and efficacy of these herbal medicines.

## Author contributions

Mohammad Abdollahi and Shirin Hasani Ranjbar gave the idea and designed the study, reviewed data, and edited the article. Zahra Jouyandeh did the search and drafted the article. All authors have read and approved content of the article.
